# Use of the Abbott i‐STAT®1 point of care device for hCG quantification in early pregnancy

**DOI:** 10.1002/ijgo.70217

**Published:** 2025-05-19

**Authors:** Caroline Joyce, Paula M. O'Shea, Rebecca Lynch, Seán J. Costelloe, Tommie V. McCarthy, John Coulter, Deirdre Hayes‐Ryan, Keelin O'Donoghue

**Affiliations:** ^1^ Pregnancy Loss Research Group, Department of Obstetrics & Gynaecology University College Cork Cork Ireland; ^2^ INFANT Research Centre University College Cork Cork Ireland; ^3^ Department of Biochemistry & Cell Biology University College Cork Cork Ireland; ^4^ Department of Clinical Biochemistry Cork University Hospital Cork Ireland; ^5^ Department of Clinical Biochemistry & Diagnostic Endocrinology The Mater Misericordiae University Hospital Dublin Ireland; ^6^ Department of Obstetrics & Gynaecology Cork University Maternity Hospital Cork Ireland; ^7^ National Perinatal Epidemiology Centre University College Cork Cork Ireland

**Keywords:** early pregnancy, human chorionic gonadotropin, point‐of‐care‐testing, pregnancy of unknown location

## Abstract

**Objectives:**

To evaluate the use of the Abbott i‐STAT®1 point‐of‐care‐test (POCT) device for measurement of human chorionic gonadotropin (hCG) to support the management of early pregnancy complications in a remote early pregnancy unit (EPU).

**Methods:**

Women attending the EPU who required an hCG blood test were invited to take part in the study. Participants provided an additional blood sample for whole blood hCG measurement using the Abbott i‐STAT®1 analyzer. The remaining sample was sent to the hospital laboratory for hCG analysis using the Abbott Architect, the designated comparator method. Statistical analysis was performed using Analyze‐IT software.

**Results:**

A total of 61 women were recruited, including cases of pregnancy of unknown location, ectopic pregnancy, and molar pregnancy. Fourteen hCG results that were outside the i‐STAT®1 assay's quantitative range, although still broadly concordant with laboratory hCG results, were excluded from statistical analysis. Analysis of the remaining 47 paired hCG results demonstrated strong agreement across the concentration range (4–2072 IU/L), with excellent Spearman correlation (*r* = 0.99, *P* < 0.001). Passing‐Bablok linear regression indicated good agreement (*y* = 1.18 + 0.96*x*) and Bland–Altman analysis showed a mean difference of −23.7 IU/L (−3.5%).

**Conclusions:**

All hCG results from the i‐STAT®1 analyzer were clinically concordant with the central laboratory method, supporting its application in an EPU setting using established clinical decision thresholds. However, further verification through larger‐scale studies is necessary before the i‐STAT®1 analyzer can be integrated into clinical practice.

## 1 INTRODUCTION

Women experiencing vaginal bleeding and/or pelvic pain in the first trimester are referred to Early Pregnancy Units (EPUs) for clinical examination and ultrasound assessment to determine pregnancy status. The differential diagnosis may encompass pregnancy of unknown location (PUL), an ongoing or failing intrauterine pregnancy (IUP), ectopic pregnancy, or molar pregnancy.^1^, ^2^ PUL describes a situation where the pregnancy test is positive, but there is no visual pregnancy on transvaginal ultrasound. With trained sonographers using high‐quality ultrasonography, the PUL rate is generally less than 15%.^3^, ^4^ PUL is a provisional diagnosis that will eventually become reclassified as an early IUP (35%–40%), failing pregnancy (44%–69%), or ectopic pregnancy (8%–14%).^5^

The National Institute for Health and Care Excellence (NICE) recommends a human chorionic gonadotropin (hCG) threshold of 1500 IU/L, above which a high‐quality ultrasound examination, performed by a trained sonographer, should detect an intrauterine pregnancy.^6^ This applies to a singleton pregnancy in a woman with a normal body mass index (calculated as weight in kilograms divided by the square of height in meters) and a normal uterus without fibroids. The provenance of hCG thresholds cited in clinical guidelines is unclear. They are likely based on older hCG radioimmunoassays, which are no longer routinely used in diagnostic laboratories and in which hCG concentrations may differ significantly from modern, high‐throughput immunoassays.^6^

Serial hCG monitoring is frequently requested in PUL, as the change in hCG concentrations over a 48‐h period can help to predict a developing ectopic pregnancy.^5^ According to clinical practice guidelines, a rise in hCG of greater than 63% within 48 h indicates an ongoing pregnancy, whereas a decline of over 50% suggests a failing pregnancy. If the change falls between these thresholds, women are classified as being at higher risk for ectopic pregnancy and require prompt medical review.^5^, ^6^ Logistic regression models can further classify PUL cases as low or high risk based on the hCG ratio between 0 and 48 h.^7^

Ectopic pregnancy is the leading international cause of death in the first trimester of pregnancy. It predominantly occurs in a fallopian tube and can result in severe complications if not promptly diagnosed and treated. Ectopic pregnancies occur in approximately 11 per 1000 pregnancies, with a maternal mortality of 0.2 per 1000 estimated ectopic pregnancies.^5^ A retrospective review of medical records for attendees to the Cork EPU in the month of July 2022 reported a PUL rate of 12% (30/249) and five of these women were diagnosed with an ectopic pregnancy.^8^ There are 10 000 ectopic pregnancies reported annually in the UK.^9^ In Ireland, the hospitalization rate for ectopic pregnancy was 17.7 per 1000 deliveries in 2016. An audit of tubal ectopic pregnancies at a major tertiary hospital in Ireland over a 3‐year period (2020–2022), identified 212 cases. Management strategies included conservative measures (*n* = 37, 17.5%), medical intervention (*n* = 74, 34.9%) and surgical procedures (*n* = 101, 47.6%).^10^ An extended audit of tubal ectopic pregnancies at this unit over a 6‐year period (2017–2022, *n* = 451) reported broadly comparable intervention rates; 22% conservative, 25% medical, and 53% surgical intervention.^11^

Blood hCG results play a crucial role in triaging women with PUL and guiding management decisions for ectopic pregnancy.^5^ Whole‐blood hCG point‐of‐care‐test (POCT) assays offer greater analytical sensitivity (2–5 IU/L) compared with qualitative urine POCT devices (20–25 IU/L).^12^ In urine, the presence of degraded free β‐hCG, known as hCG beta core fragment (hCGβcf) can lead to false‐negative results due to the high dose hook effect.^13^ Quantitative blood hCG POCT assays provide a clinical decision threshold of 5 IU/L for early pregnancy detection, offering improved sensitivity in healthcare settings.

In remotely located EPUs without on‐site access to laboratory hCG testing, blood hCG POCT results could support clinical decision making. This may improve integrated care and resource efficiency, by reducing delays in blood hCG results, while also alleviating the distress and anxiety that some women experience when attending a maternity hospital for suspected pregnancy loss.^14^ This study, aimed to evaluate the Abbott i‐STAT®1 POCT analyzer based on key performance criteria, including patient comparisons, assay imprecision, and suitability for clinical use.

## 2 MATERIALS AND METHODS

### 2.1 Study design

All women attending the EPU in Cork University Maternity Hospital between January and June 2023, who required an hCG blood test for scheduled care, were invited to participate in the study. To ensure an adequate sample size for verification studies, women with PUL as well as those undergoing follow up for ectopic or molar pregnancies, were also recruited. After obtaining written consent, an additional lithium heparin blood sample was collected for POCT hCG analysis. Whole‐blood total β‐hCG was measured on the Abbott i‐STAT®1 analyzer in the EPU and the remaining sample was sent to the central laboratory for analysis of plasma total β‐hCG on the Abbott Architect (Abbott Diagnostics, IL, USA) analyzer. It should be noted that hCG results measured on the i‐STAT®1 analyzer were not used to inform patient management during the study. Written informed consent was obtained from all patients participating in this study in accordance with ethical approval and the study was approved by the Cork Research Ethics Committee (ECM4 (b) 01/11/2022 & ECM5 (8) 05/10/2022 & ECM3 (ss) 28/03/23).

### 2.2 Analytical methods

The Abbott i‐STAT®1 (Abbott Point of Care Inc., USA) is a handheld analyzer that is CE (Conformité Européenne) marked in compliance with the European In‐Vitro Diagnostic Regulation 2017/746. It measures total β‐hCG in a dry reagent cartridge (reference 05P58‐25) and reports a quantitative result in the linear range of the assay (5–2000 IU/L) and a qualitative result outside these thresholds (Table 1). Whole blood was loaded directly from the specimen tube using the i‐STAT®1 dispensing tip (reference 06F24‐20) and the test was performed according to the manufacturer's instructions.^15^ Internal laboratory controls using low (Abbott level 1, reference 05P59‐01) and high (Abbott level 3, reference 05P59‐03) hCG concentrations were analyzed on alternate weeks during the study period. Levels were chosen to assess assay performance at the critical clinical decision thresholds. Accuracy of the i‐STAT®1 analyzer was further assessed using samples from the Welsh External Quality Assessment Scheme (WEQAS).

**TABLE 1:** Comparison of point‐of‐care device to central laboratory analyzer.

Parameters	Abbott i‐STAT®1	Abbott Architect
Sample type	Plasma LH	Serum, Plasma LH
CE marked	Yes	Yes
Minimum volume, μL	17	75
Linear range, IU/L	5–2000	1.2–15 000
Hook effect, IU/L	>300 000^a^	N/A
Assay time, min	10	20
Total TAT, min	15	120
hCG isoforms	Total β‐hCG^b^	Total β‐hCG^b^
Test principle	2‐step ELISA/ECD	2‐step CMIA/LUM
WHO IS	5th (07/364)	4th (75/589)

Abbreviations: CE, Conformité Européenne; CMIA, chemiluminescent microparticle immunoassay; ECD, electrochemical detection; ELISA, enzyme‐linked immunosorbent assay; LH, lithium heparin; LUM, luminometer; N/A, not available; TAT, turn‐around‐time (sample collection to result), WHO IS, World Health Organization International Standard.

^a^ Sowder et al.^19^ reported a higher hook effect of 400 000 IU/L.

^b^ Intact hCG and free β subunit.

Study plasma samples were analyzed in the core laboratory on the Abbott Architect analyzer using a total β‐hCG assay, according to the manufacturer's instructions.^16^ This assay is accredited to ISO15189 (2012) and was designated the comparator assay for accuracy assessment in this study. Liquid quality control material (Technopath Multichem 1A, reference O5P76–10) and External Quality Assessment (EQA) samples were analyzed throughout the study period.

### 2.3 Statistical analysis

Statistical analysis was performed using Microsoft Excel 2021 and Analyze‐IT software. Parametric testing of the data was performed using the Shapiro–Wilk test, and descriptive statistics were performed on the baseline study characteristics. Continuous parametric data were represented as mean and standard deviation (SD) and non‐Gaussian data, as median and interquartile range. Whole blood β‐hCG results from the i‐STAT®1 were compared with those from the Abbott Architect analyzer using Spearman's correlation coefficient, with a *P* value less than 0.05 considered statistically significant. Passing‐Bablok linear regression analysis and Bland–Altman difference plots were used to assess method agreement and bias of the different assays respectively, in accordance with the Clinical Laboratory Standards Institute (CLSI) EP09‐A3 guideline.^17^

## 3 RESULTS

### 3.1 Study characteristics

In total, 61 women were recruited to the study including women with PUL, suspected ectopic pregnancies, and confirmed molar pregnancy. Some cases of PUL evolved into viable/nonviable IUP with the cohort ultimately consisting of women with intrauterine pregnancy/pregnancy of uncertain viability (*n* = 10, 16.4%), PUL (*n* = 9, 14.8%), ectopic pregnancy (*n* = 8, 13.1%), miscarriage (*n* = 29, 47.5%), molar pregnancy (*n* = 3, 4.9%), and retained pregnancy tissue (*n* = 2, 3.3%). The three participants undergoing follow up for molar pregnancy had their results (<5, 13, and 23 IU/L) confirmed using a CE‐marked total β‐hCG assay for oncology (Cobas® Elecsys). The median age of study participants was 33 years (interquartile range, 30–36 years) with a minimum to maximum maternal age of 20–40 years. The mean gestational age was 7 weeks (±2.4 SD), covering periods when the gestational sac (5 weeks), fetal pole (~6 weeks), and fetal heartbeat (~6–7 weeks) are typically visible on transvaginal ultrasound examination.^18^

### 3.2 Statistical analysis

**FIGURE 1:**
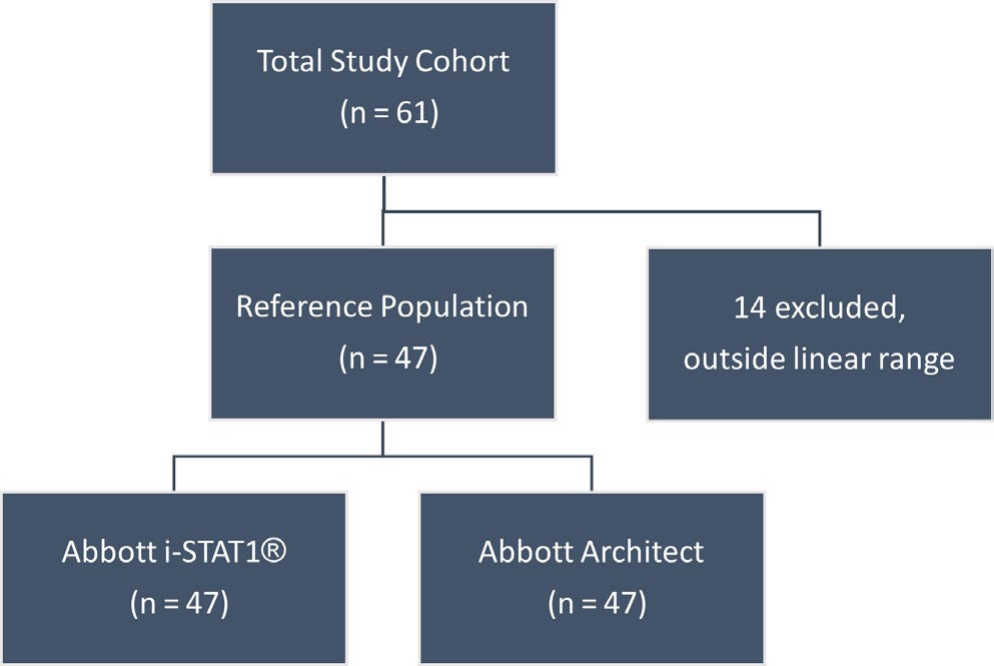
Recruitment schematic.

**FIGURE 2:**
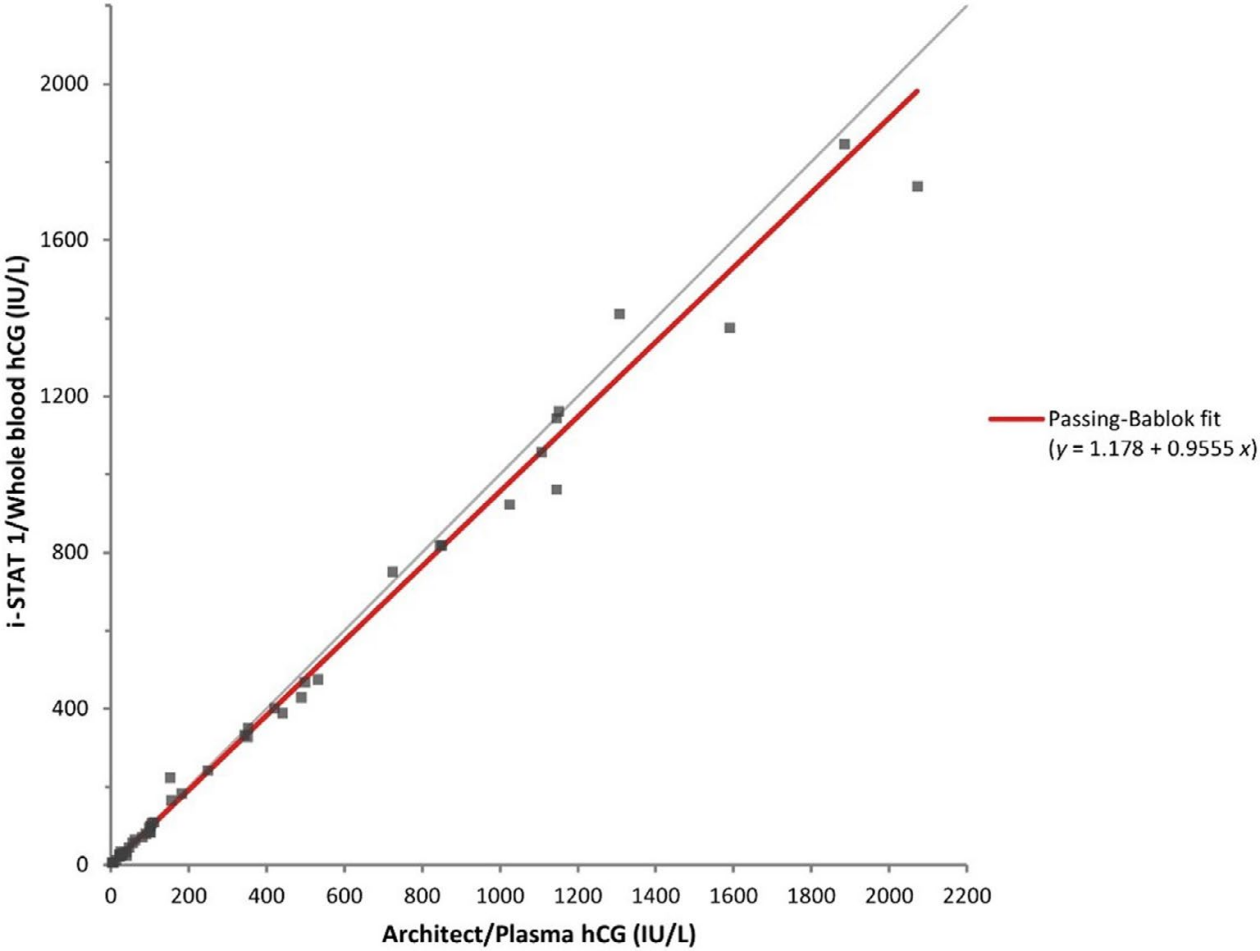
Method comparison i‐STAT®1 versus Architect: Passing‐Bablok regression line.

**FIGURE 3:**
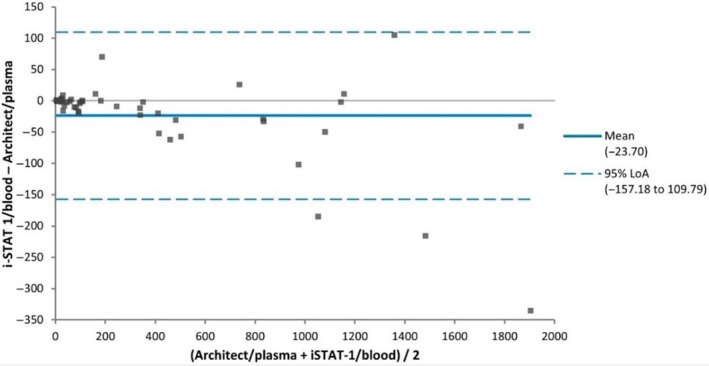
Method comparison i‐STAT®1 versus Architect: Bland–Altman difference plot.

The Shapiro–Wilk test demonstrated that hCG results from the i‐STAT®1 were not normally distributed (W statistic: 0.78, *P* < 0.001). POCT results outside the linear range of the i‐STAT®1 assay (*n* = 14, 23%) were excluded from statistical analysis, despite being clinically consistent with the central laboratory assay (see Figure 1 and Table 2). The β‐hCG results from the Abbott i‐STAT®1 demonstrated excellent agreement with those from the Abbott Architect across the hCG concentration range (4–2072 IU/L). Spearman correlation showed *r* = 0.995 (*P* < 0.001). Passing‐Bablok regression yielded *y* = 1.18 + 0.96*x*, whereas the Bland–Altman difference plot indicated a mean absolute difference of −23.7 IU/L and percentage difference of −3.5% (see Table 3 and Figures 2 and 3). Three of the six EQA samples fell outside the assay's measuring range (<5, >2000 IU/L); however, all qualitative results were correct. The remaining EQA samples were within acceptable limits, with values falling within two SD of the WEQAS target mean (see Table 4). The i‐STAT®1 assay demonstrated acceptable precision, with controls within their assigned range and coefficients of variation (CV) meeting the manufacturer's targets (Level 1: 24.7, CV 15.8% and Level 2: 1571.5, CV 17.0%). The Abbott Architect assay also showed acceptable precision across all three control levels (Level 1: 11.3%, Level 2: 4.2%, Level 3: 2.7%).

**TABLE 2:** Specimens outside the linear range of the POCT hCG assay.

Clinical details	Abbott Architect	i‐STAT®1
Ectopic	12 388	>2000
Miscarriage	40 349	>2000
IPUV	101 664	>2000
IPUV	219 759	>2000
IPUV	2600	>2000
Miscarriage	3041	>2000
IPUV	5270	>2000
IPUV	6897	>2000
Miscarriage	<1.2	<5.0
Miscarriage	2526	>2000
Miscarriage	3033	>2000
Miscarriage	3949	>2000
Molar pregnancy	<1.2	<5
RPT	7323	>2000

Abbreviations: hCG, human chorionic gonadotropin; IPUV, intrauterine pregnancy of uncertain viability; POCT, point‐of‐care‐test; RPT, retained pregnancy tissue.

**TABLE 3:** Method comparison—Statistical assessment of agreement between the i‐STAT®1 POCT device and the Abbott Architect (Designated comparator) method.

Designated comparator method	POCT device
Abbott Architect, total β‐hCG	Abbott i‐STAT®1 (*n* = 47)
Passing‐Bablok Regression	*y* = 1.18 + 0.96*x*
Intercept (95% CI)	1.18 (−1.15 to 3.40)
Slope (95% CI)	0.96 (0.90–0.99)
Spearman correlation	*R* = 0.995, *P* < 0.001
Bland–Altman Difference plot	Mean difference, IU/L
Mean difference (95% CI)	−23.7 (−43.7 to −3.70)
Lower LOA (95% CI)	−157.2 (−191.6 to −122.8)
Upper LOA (95% CI)	109.8 (75.4–144.2)
Bland–Altman % Difference plot	Mean difference, %
Mean % difference (95% CI)	−3.52 (−7.7 to 0.67)
Lower LOA (95% CI)	−31.5 (−38.7 to −24.2)
Upper LOA (95% CI)	24.4 (17.2–31.6)

Abbreviations: CI, confidence interval; LOA, limits of agreement; POCT, point‐of‐care‐test.

**TABLE 4:** Accuracy verification for i‐STAT®1.

WEQAS distribution, sample number	i‐STAT®1 target mean hCG, IU/L	i‐STAT®1 method SD	i‐STAT®1 EPU mean hCG, IU/L
WS0722, sample 1	46.08	5.83	44.1
WS0722, sample 2	>2000	NA	>2000
WS0722, sample 3	1433.5	36.37	1347.2
WS0922, sample 1	>2000	NA	>2000
WS0922, sample 2	<5	NA	<5
WS0922, sample 3	450.62	18.68	421.3

Abbreviations: EPU, early pregnancy unit; NA, not available; WEQAS, Welsh External Quality Assessment Scheme.

## 4 DISCUSSION

The Abbott i‐STAT®1 POCT device and the Abbott Architect core laboratory analyzer demonstrated excellent agreement between total β‐hCG blood results with a minimal negative proportional bias of 4%. This confirms the reliability of hCG results at the clinical decision threshold (1500 IU/L) for PUL, highlighting the i‐STAT®1 analyzer's potential to support clinical management in the EPU.^3^, ^6^ Implementing this service would be particularly beneficial in EPUs without on‐site laboratory access or where delays in obtaining hCG results impact patient care. The availability of POCT blood hCG analysis could facilitate rapid triage of women with PUL into high‐ and low‐risk groups using decision support algorithms.

During the study period, 47 of the 61 participants (77%) had hCG values below 2000 IU/L, supporting the view that most hCG results in PUL triage reside within the 1000–2000 IU/L range, which aligns with the quantitative range of the i‐STAT®1. This further supports the use of the Abbott i‐STAT®1 device for blood hCG testing to guide management in this clinical context. In most cases of ectopic pregnancy following a PUL classification, initial hCG concentrations are typically <1000 IU/L.^5^ However, once an ectopic pregnancy is confirmed by ultrasound examination, serial hCG measurements are no longer necessary. In such cases, hCG results can assist in determining the appropriate approach for conservative, medical, or surgical management. We do not recommend point‐of‐care hCG testing for the serial monitoring of medically managed ectopic pregnancies. In these situations, use of a central laboratory hCG assay is preferable to ensure continuity and accuracy, especially as patients may present outside regular hours and require assessment across different healthcare settings.

Currently available POCT devices for near‐patient blood hCG testing include the Abbott i‐STAT®1, Radiometer AQT90 FLEX®, and VEDLAB Easy Reader.^2^, ^19^, ^20^, ^21^, ^22^, ^23^ In a separate study, the Abbott i‐STAT®1 POCT device correlated well with the Beckman Coulter DXi800 hCG assay in an antenatal clinic setting for confirming or excluding early pregnancy (≤6 weeks of pregnancy).^20^ The AQT90, a benchtop POCT device, offers a broader measuring range (2–5000 IU/L) compared with the Abbott i‐STAT®1, potentially extending its clinical utility. However, as shown in this study and corroborated by Kyriacou et al.,^1^ only a minority of patients (19.6%) triaged for PUL have hCG concentrations greater than 2000 IU/L, suggesting that the i‐STAT®1 range is sufficient for most cases.

A limitation of our study was the absence of extremely high hCG values to assess the potential hook effect threshold of the POCT assay. The hook effect occurs when hCG concentrations become so elevated that they saturate the anti‐hCG antibody, leading to false‐negative results. This phenomenon can delay diagnosis or result in missed diagnoses with potentially adverse outcomes. Although the highest hCG result assessed in our study was 219 759 IU/L, it was below the manufacturer's stated hook effect threshold for the i‐STAT®1 (300 000 IU/L). Other studies suggest that the hook effect could occur at hCG levels as high as 400 000 IU/L for this device.^19^ As a result of its limited measuring range, POCT is unsuitable for monitoring total β‐hCG in advanced ectopic pregnancies or suspected molar pregnancies, where very high hCG levels may exceed the assay's detection limits or lead to falsely low results because of the high dose hook effect. Furthermore, the Abbott i‐STAT®1 total β‐hCG assay is CE‐marked for early pregnancy detection and monitoring only, and is not approved for use in oncology settings, such as trophoblastic disease or molar pregnancy.

The primary advantage of POCT devices is their ability to obtain hCG results within 20 min, facilitating faster decision making and improving patient triage for timely care during clinic visits. A key strength of the Abbott i‐STAT®1 POCT device is its portability as a handheld unit, allowing easy transfer between consultation rooms. Its user‐friendly interface and minimal maintenance requirements help to reduce the likelihood of errors, making it accessible for healthcare professionals outside the laboratory. Furthermore, the i‐STAT®1 is the only hCG POCT device included in the WEQAS POCT external quality assessment scheme, ensuring continuous monitoring of its reliability and accuracy in clinical practice.

Integrating a blood hCG POCT device into the EPU care pathway could improve triage for women at risk of pregnancy complications by applying established clinical decision thresholds for PUL, potentially reducing unnecessary hospital admissions. However, current national and international clinical guidelines do not recommend using hCG POCT results for PUL management. Although some maternity hospitals lack on‐site laboratory access, serial hCG measurements using standard laboratory blood tests remain the reference standard for assessing early pregnancy.^2^, ^6^ Further research is needed to evaluate the suitability of existing hCG decision thresholds for POCT devices and to compare their performance with commercially available immunoassays used in central laboratories. Moreover, POCT could offer a highly sensitive hCG test in community settings, offering significant benefits for managing early medical abortion.^24^, ^25^

This study contributes to the limited body of evidence comparing whole blood POCT hCG results with traditional laboratory‐based testing. Although the current i‐STAT®1 assay upper quantification limit is 2000 IU/L, several next‐generation POCT platforms now quantify up to 5000 IU/L. Our protocol and performance data therefore provide a foundation for wider adoption as the technology evolves. Larger‐scale studies are needed to evaluate the clinical impact of POCT hCG results on PUL triage protocols. Furthermore, a comprehensive cost–benefit analysis is essential to assess the economic implications of rapid POCT hCG results in the EPU, particularly with regard to hospital admissions, length of stay, and other factors, similar to evaluations conducted in emergency medicine settings.^21^

In conclusion, the Abbott i‐STAT®1 POCT device demonstrated strong performance compared with the main laboratory immunoassay for quantifying hCG in early pregnancy. Implementing POCT for blood hCG measurement could improve efficiency in the EPU and contribute to safer clinical outcomes by enabling prompt decisions regarding the diagnosis or exclusion of ectopic pregnancy. The i‐STAT®1 hCG POCT device could assist clinicians in triaging patients based on clinical decision thresholds, guiding management of suspected PUL or ectopic pregnancies. However, before this POCT device could be recommended for routine clinical use in early pregnancy, a larger‐scale prospective verification study with an expanded cohort of women is necessary.

## AUTHOR CONTRIBUTIONS

CMJ, PMOS, DHR, and KOD designed the study and drafted the manuscript; CMJ and RL collected the data; CMJ and PMOS performed the statistics; CMJ, POS, DHR, SC, JC, TMcC, and KOD approved the final version of the manuscript. KOD is the guarantor.

## CONFLICT OF INTEREST STATEMENT

The authors have no conflicts of interest.

## Data Availability

In accordance with the journal's guidelines, we will provide our data for independent analysis by a selected team or by the Editorial Team for the purposes of additional data analysis or for the reproducibility of this study in other centers if such is requested.
